# Inferring bovine tuberculosis transmission between cattle and badgers via the environment and risk mapping

**DOI:** 10.3389/fvets.2023.1233173

**Published:** 2023-09-22

**Authors:** You Chang, Nienke Hartemink, Andrew W. Byrne, Eamonn Gormley, Guy McGrath, Jamie A. Tratalos, Philip Breslin, Simon J. More, Mart C. M. de Jong

**Affiliations:** ^1^Quantitative Veterinary Epidemiology Group, Wageningen University and Research Centre, Wageningen, Netherlands; ^2^Biometris, Wageningen University and Research Centre, Wageningen, Netherlands; ^3^One-Health and Welfare Scientific Support Unit, Department of Agriculture, Food and the Marine, National Disease Control Centre, Dublin, Ireland; ^4^Tuberculosis Diagnostics and Immunology Research Centre, School of Agriculture, Food Science, and Veterinary Medicine, College of Life Sciences, University College Dublin, Dublin, Ireland; ^5^Centre for Veterinary Epidemiology and Risk Analysis, School of Veterinary Medicine, University College Dublin, Belfield, Dublin, Ireland; ^6^Ruminant Animal Health Division, Department of Agriculture, Food and the Marine, Dublin, Ireland

**Keywords:** bovine tuberculosis, environmental transmission, domestic wildlife interface, R map, next-generation matrix method

## Abstract

Bovine tuberculosis (bTB), caused by *Mycobacterium bovis*, is one of the most challenging and persistent health issues in many countries worldwide. In several countries, bTB control is complicated due to the presence of wildlife reservoirs of infection, i.e. European badger (*Meles meles*) in Ireland and the UK, which can transmit infection to cattle. However, a quantitative understanding of the role of cattle and badgers in bTB transmission is elusive, especially where there is spatial variation in relative density between badgers and cattle. Moreover, as these two species have infrequent direct contact, environmental transmission is likely to play a role, but the quantitative importance of the environment has not been assessed. Therefore, the objective of this study is to better understand bTB transmission between cattle and badgers via the environment in a spatially explicit context and to identify high-risk areas. We developed an environmental transmission model that incorporates both within-herd/territory transmission and between-species transmission, with the latter facilitated by badger territories overlapping with herd areas. Model parameters such as transmission rate parameters and the decay rate parameter of *M. bovis* were estimated by maximum likelihood estimation using infection data from badgers and cattle collected during a 4-year badger vaccination trial. Our estimation showed that the environment can play an important role in the transmission of bTB, with a half-life of *M. bovis* in the environment of around 177 days. Based on the estimated transmission rate parameters, we calculate the basic reproduction ratio (R) within a herd, which reveals how relative badger density dictates transmission. In addition, we simulated transmission in each small local area to generate a first between-herd R map that identifies high-risk areas.

## 1. Introduction

Bovine tuberculosis (bTB) is one of the most complicated, persistent, and expensive health issues globally. While its primary impact is on bovines, it can infect many other mammals, including humans and wildlife animals (1). bTB is very persistent in livestock globally due to the involvement of several wildlife species in bTB transmission. Notable examples include badgers in the UK and Ireland, brushtail possums in New Zealand, wild boars in Spain (2), red deer in Austria (3), and African buffalo in South Africa (4). Although pasteurization of milk can reduce human infection, *Mycobacterium bovis* is estimated to cause ~10% of total human TB cases in developing countries (5, 6). The impact of bTB extends beyond public health with substantial economic consequences, costing approximately US$3 billion globally (7). In the Republic of Ireland (bTB) alone, more than 15,000 cattle have been removed annually over the last decade. In 2020, the total programme expenditure cost was €97 million and is rising year-on-year (8).

The Irish national bTB eradication programme is underpinned by a test-and-removal strategy, leading to the slaughter of all cattle that are positive to the single intradermal comparative tuberculin test (SICTT), performed at least annually in each Irish herd (9). This strategy has been successful in eradicating bTB in some countries, such as Australia and some northern European countries (10). In Ireland, however, progress has stalled in the national eradication programme (11, 12), at least in part due to the presence of other reservoirs of infection, including badgers (*Meles meles*; 13). Badger vaccination has proven effective at reducing badger susceptibility, both in pen and field studies (13–15), and a badger vaccination programme is now being progressively incorporated into a national programme (16, 17).

A number of different approaches have been used in recent studies to investigate the role of badgers in bTB transmission and persistence. In Republic of Ireland (ROI), badger culling trials resulted in a significant decrease in cattle incidence in areas of badger culling compared to reference areas (13, 18, 19). In Britain, the Randomized Badger Cull Trial (RBCT) found evidence for decreased risk of bTB breakdown in proactive cull areas; however, *post-hoc* analysis suggested that a transitory increased risk to neighboring areas could occur (20). Using a case–control design, badger relative abundance in the vicinity of cattle herds was identified as an important risk factor for bTB herd breakdown risk in Britain (21) and Ireland (22). In addition, studies of road-killed badgers found strong evidence that badgers and cattle are colonized by the same *M. bovis* strain in the same area (23, 24). Most recently, genomic epidemiology has been used to understand transmission direction between species, generally suggesting that within-species transmission is more common than between-species transmission in study areas (25–28). The relative importance of cattle and badgers appears to be context specific (26, 28–30). Although these studies provide important insight that badger bTB is associated with cattle bTB, a quantitative understanding of how relative badger density impacts bTB transmission in this cattle and badger episystem is still lacking.

The main transmission routes of bTB are believed to be droplets, aerosols, and fecal to oral transmission (31). These three transmission mechanisms are intrinsically similar, involving an environmental vehicle such as droplets, aerosols, feces, urine, etc. *M. bovis*-laden droplets and aerosols may also settle onto pastures and contribute to the subsequent environment for oral transmission. The distinction between these transmission routes lies in the duration between the shedding moment and the time point of inhaling or ingesting *M. bovis*. Buddle et al. (32) have proposed a role for environmental transmission as an explanation for the variable efficacy observed in an overview of vaccine trials for the control of tuberculosis in cattle, wildlife, and humans. *Mycobacterium tuberculosis* complex (MTBC) has been demonstrated to be present at the wildlife–environment–livestock interface in Spain (33) and Italy (34), and more specifically, *M. bovis* has been detected in badger feces in the UK (35) and experimentally infected cattle (36). In recent global positioning system (GPS) studies, badgers barely have direct contact with cattle, suggesting that environmental transmission may indeed play an important role in bTB transmission (37, 38). However, to this point, the quantitative importance of bTB transmission via the environment has barely been considered (39).

Therefore, this study aims to gain a better understanding of the quantitative role of badgers and cattle in TB transmission via environmental transmission and quantify the impact of relative badger density on bTB transmission in a spatial context. With this information, we can identify high-risk areas for transmission where bTB might sustain locally and assess whether badger vaccination along with the test-and-removal strategy is sufficient to control transmission in different areas.

## 2. Materials and methods

In this study, we aim to understand the local transmission of bTB in a cattle and badger system. To this end, we develop an environmental transmission model that incorporates both within-herd/territory transmission and between-species transmission.

In Section 2.1, we present the structure of an environmental transmission model for the cattle and badger system. The model parameterisation, which is partially drawn from existing literature, is described in Section 2.2, and the estimation of transmission and decay rate parameters from time-series infection data is presented in Section 2.3. The infection data used in the estimation are explained in Section 2.4. With the estimated parameters, we use the next-generation matrix (NGM) method to calculate the basic reproduction ratio for the within-herd transmission and investigate the impact of the relative badger density on the within-herd R (Section 2.5.1). Furthermore, we use simulation to generate between-herd R maps (Section 2.5.2).

### 2.1. Model description

We developed a stochastic compartmental model with environmental transmission for a cattle and badger system. In this system, a herd of cattle and a social group of badgers refer to the animals of interest, whereas a farm and a badger territory each refer to a spatial unit. A farm is a spatial location for a herd, with all cattle in the herd registered to the same herd identifier. In Ireland, a farm can consist of several fragments of land that can be spatially dispersed, and we assume that cattle spend time on each fragment proportionally to its area. A badger territory is an area where a social group of badgers primarily resides, which usually contains a main sett and several outlier setts. The model incorporates a geographic overlay of these two spatial units, where the between-species transmission and the spatial spread are assumed to occur.

#### 2.1.1. A completely shared area with one farm and one badger sett territory

**Figure 1:**
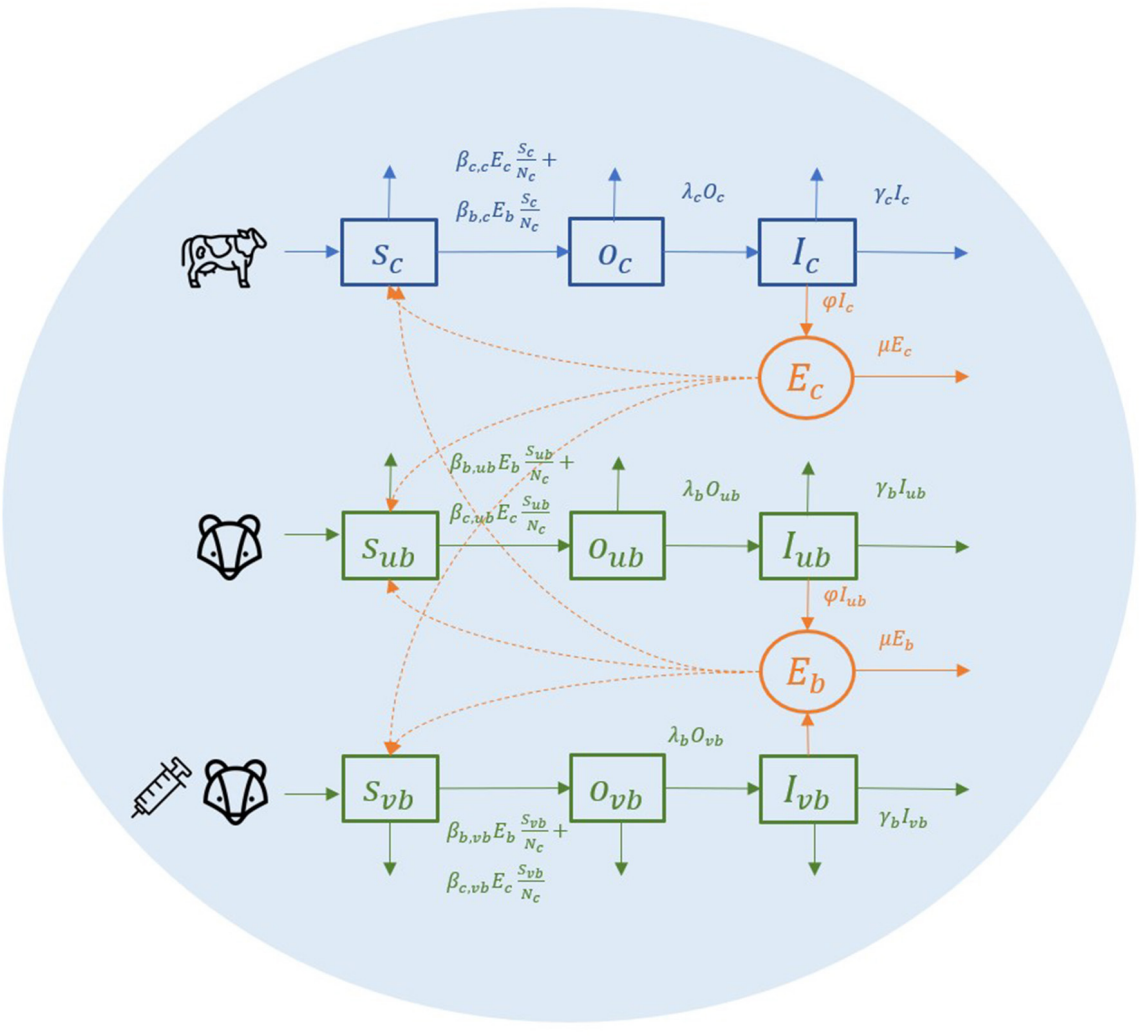
A conceptual diagram of within-herd/territory transmission in a completely shared area with one farm and one badger sett territory.

To explain this environmental transmission model, we first look at a conceptual spatial structure in a small local area where one farm and one badger territory are completely overlapping (Figure 1). In this local area, individual badgers from one social group and individual cattle from one herd share the same environment (light blue circle in Figure 1). Cattle, unvaccinated badgers, and vaccinated badgers are the three types of animals in the model, abbreviated as c, ub, and vb in subscripts. Vaccinated and unvaccinated badgers can exist in the same area because of the ongoing vaccination programme, and they are assumed to differ in terms of susceptibility but not infectivity (15). All individual animals are classified into three compartments: susceptible (S), latent (O), and infectious (I). Susceptible individuals can get infected by the same species or another species at a certain transmission rate after being exposed to *M. bovis*. When infection becomes established, animals can become infectious, although the length of the latent period is controversial. Infectious animals can shed *M. bovis* into the environment of their spatial units. We assume that *M. bovis* in the environment (denoted as *E*_*c*_, *E*_*b*_) are distributed evenly in the farm and the badger territory, which is the same area in this example (light blue circle in Figure 1). Since the vaccination is assumed not to reduce badgers' infectivity (15), the amount of *M. bovis* shed by infectious badgers is represented by compartment *E*_*b*_, regardless of whether the infectious badgers are vaccinated or unvaccinated.

The transmission rate from cattle to cattle is $\beta_{c,c}S_{c}\frac{E_{c}}{N_{c}}.\;$The β_*c, c*_ represents the cattle transmission rate parameter per contact with one unit of *E*_*c*_ per day. Here, we use cattle number *N*_*c*_ to represent the area size, hence for each susceptible bovine, the probability that the contact with *E*_*c*_ is made is equal to $\frac{E_{c}}{N_{c}}$. The same rules apply to all the other transmission rates. For example, the transmission rate from badger to cattle is $\beta_{b,c}S_{c}\frac{E_{b}}{N_{c}}\;$in which the probability that the contact with *E*_*b*_ is made for each susceptible badger is $\frac{E_{b}}{N_{c}}$. We use one denominator in both cattle and badgers to have a unified representation of the area in this two-host system. In transmission rate parameter β_*b,ub*_ and β_*b,vb*_, we do not distinguish whether the infection source badger is vaccinated or unvaccinated (the first b of the subscript) because the vaccination is assumed not to reduce the infectivity, and environmental contamination from the vaccinated or unvaccinated badgers is not distinguished in *E*_*b*_.

Infected animals (O compartment) can develop further into infectious state (I compartment) at a rate of λ_*c*_*I*_*c*_ and λ_*b*_*I*_*b*_. Infectious animals are removed at a rate of α_*c*_*I*_*c*_ and α_*b*_*I*_*b*_, caused by cattle test-and-removal and by bTB-induced badger death, respectively. We assume the background death rate parameters are equal to the birth rate of animals (α_*c*_, α_*b*_) and that all newborn animals are susceptible.

Infectious animals can shed *M. bovis* into the environment, where it subsequently decays. The shedding and decay of *M. bovis* are modeled deterministically as follows:

(1) $$\begin{array}{l} \frac{dE_{c\left(i\right)}}{dt}=\varphi I_{c\left(i\right)}-\mu E_{c\left(i\right)} \end{array}$$

(2) $$\begin{array}{l} \frac{dE_{b\left(j\right)}}{dt}=\varphi I_{ub\left(j\right)}+\;\varphi I_{vb\left(j\right)}-\mu E_{b\left(j\right)} \end{array}$$

where *i, j* denote the index for farm and badger territories, respectively. We assume that the decay of *M. bovis* has the same decay rate parameter μ despite the different infection source and strains (μ*E*_*c*_ for cattle and μ*E*_*b*_ for badgers). The shedding rate parameter φ is scaled as a function of the decay rate parameter ($\frac{\mu^{2}}{-1+e^{-\mu }+\mu }$) (40). The reason for this scaling is that the shedding rate parameter φ and the transmission rate parameter β are structurally not jointly identifiable from infection data (41). Therefore, we choose to fix the shedding rate parameters and estimate the different transmission rate parameters from infection data (more details in Eqs. 5, 6). With the standardization ($\varphi =\frac{\mu^{2}}{-1+e^{-\mu }+\mu }$), the transmission rate parameters represent the transmission rate from one typical infectious individual to a susceptible individual during one interval starting in a clean environment (40).

#### 2.1.2. Many farms and many badger territories that partially overlap

**Figure 2:**
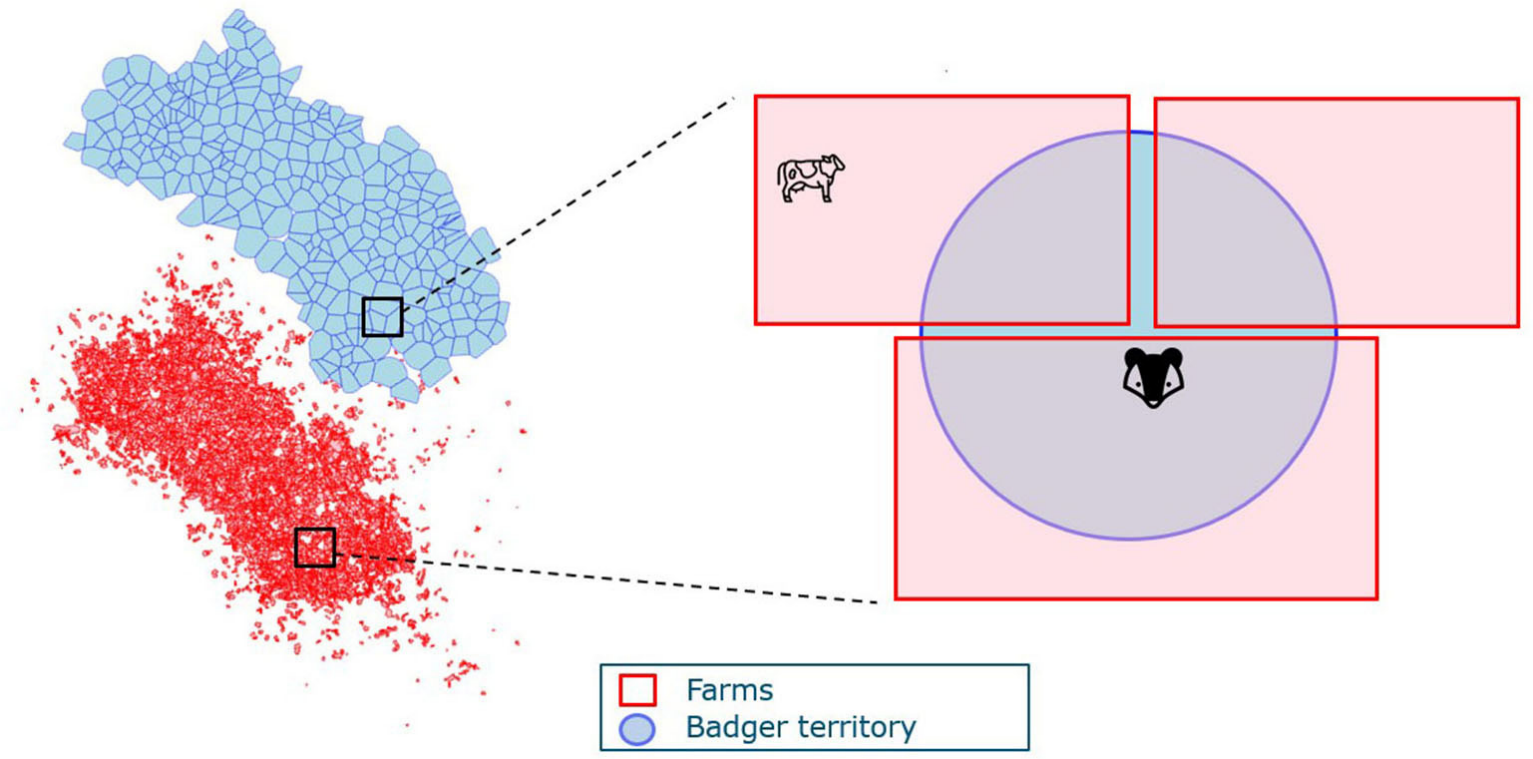
An example of the spatial structure of farms and badger territories. The blue map represents badger territories, and the red irregular shapes delineate farm boundaries.

We then consider the spatial structure of badger territories and farms in the full model. Badger territories can overlap with several farms; hence, badgers act as vectors that facilitate between-herd transmission. Similarly, herds can overlap with several badger territories and facilitate transmission between different badger social groups (Figure 2). To account for the spatial structure in the model, the exposure from the other species is weighted by the ratio of (the total area of overlap between farms and badger territories) and (the total area of farms or badger territories). The denominator in the transmission rate for badgers is also adjusted with the weighted cattle number as a representation of the badger territory area. The ordinary differential equation version of the transmission is presented in Eqs. 3, 4.

(3) $$\begin{array}{l} \frac{dO_{c\left(i\right)}}{dt}=\beta_{c,c}S_{c\left(i\right)}\frac{E_{c\left(i\right)}}{N_{c\left(i\right)}}\;+\beta_{b,c}S_{c\left(i\right)}\frac{\sum_{j=1,..k}E_{b\left(j\right)}\frac{A_{\left(ij\right)}}{AT_{\left(j\right)}}}{N_{c\left(i\right)}}-\lambda_{c}O_{c\left(i\right)} \end{array}$$

(4) $$\begin{array}{l} \frac{dO_{b\left(j\right)}}{dt}=VC(\beta_{b,vb}S_{vb\left(j\right)}\frac{E_{b\left(j\right)}}{\sum_{i=1..m}N_{c\left(i\right)}\frac{A_{\left(ij\right)}}{AF_{\left(i\right)}}}\\ +\beta_{c,vb}S_{vb\left(j\right)}\frac{\sum_{i=1,..m}E_{c\left(i\right)}\frac{A_{\left(ij\right)}}{AF_{\left(i\right)}}}{\sum_{i=1..m}N_{c\left(i\right)}\frac{A_{\left(ij\right)}}{AF_{\left(i\right)}}})\\ +\left(1-VC\right)(\beta_{v,ub}S_{ub\left(j\right)}\frac{E_{b\left(j\right)}}{\sum_{i=1..m}N_{c\left(i\right)}\frac{A_{\left(ij\right)}}{AF_{\left(i\right)}}}\\ +\beta_{c,ub}S_{ub\left(j\right)}\frac{\sum_{i=1,..m}E_{c\left(i\right)}\frac{A_{\left(ij\right)}}{AF_{\left(i\right)}}}{\sum_{i=1..m}N_{c\left(i\right)}\frac{A_{\left(ij\right)}}{AF_{\left(i\right)}}})-\lambda_{b}O_{b\left(j\right)} \end{array}$$

Farms and badger territories are the two spatial units in the model where *i, j* denote the index for farm and badger territories, respectively. *A*_(*ij*)_ denotes the total area of overlap between farm *i* and territory *j*. $\frac{A_{\left(ij\right)}}{AF_{\left(i\right)}}$ represents the proportion of farm *i* that overlaps with territory *j*. Similarly, $\frac{A_{\left(ij\right)}}{AT_{\left(j\right)}}$ is the proportion of territory *j* that overlaps with farm *i*.

Cattle on farm *i* can get infected by *M. bovis* on the farm excreted by cattle (*E*_*c*(*i*)_) at rate $\beta_{c,c}S_{c\left(i\right)}\frac{E_{c\left(i\right)}}{N_{c\left(i\right)}}$ or excreted by badgers whose territories overlap with the farm *i* at rate $\beta_{b,c}S_{c\left(i\right)}\frac{\sum_{j=1..k}E_{b\left(j\right)}\frac{A_{\left(ij\right)}}{AT_{i}}}{N_{c\left(i\right)}}$. Multiple badger territories (*j* = 1..*k*) can overlap with farm *i*, so the contribution from these territories (*j* = 1..*k*) are summed. For each territory j, only the part of the territory that is located inside farm *i* can pose a threat on infecting cattle; hence, each *E*_*b*(*j*)_ is adjusted to $E_{b\left(j\right)}\frac{A_{\left(ij\right)}}{AT_{i}}$.

Similarly, badgers can get infected by badgers in their own territory *j* or by cattle in farms that overlap with *j*. As mentioned in Section 2.1.1, we use a unified representation of the area, namely the number of cattle in that area. Therefore, the area of badger territory is represented by the weighted number of cattle as $\sum_{i=1..m}N_{c\left(i\right)}\frac{A_{\left(ij\right)}}{AF_{\left(i\right)}}$, as territory j overlaps with different farms (*i* = 1..*m*). A proportion of the badgers are vaccinated, denoted as *VC* (vaccination coverage). Vaccinated badgers are assumed to have reduced susceptibility but the same infectivity as the unvaccinated badgers. Therefore, transmission from infectious badgers to vaccinated badgers is modeled as ${\left(VC\right)\beta }_{b,vb}S_{vb\left(j\right)}\frac{E_{b\left(j\right)}}{\sum_{i=1..m}N_{c\left(i\right)}\frac{A_{\left(ij\right)}}{AF_{\left(i\right)}}}$ and transmission from infectious badgers to unvaccinated badgers as $\left(1-VC\right)\beta_{b,ub}S_{ub\left(j\right)}\frac{E_{b\left(j\right)}}{\sum_{i=1..m}N_{c\left(i\right)}\frac{A_{\left(ij\right)}}{AF_{\left(i\right)}}}$. For cattle-to-badger transmission, only part of farm *i* is located inside the badger territory j, so *E*_*c*(*j*)_ is adjusted with $E_{c\left(j\right)}\frac{A_{\left(ij\right)}}{AF_{i}}$. Therefore, the cattle-to-badger transmission rate is denoted as $\left(VC\right)\beta_{c,vb}S_{vb\left(j\right)}\frac{\sum_{i=1,..m}E_{c\left(i\right)}\frac{A_{\left(ij\right)}}{AF_{\left(i\right)}}}{\sum_{i=1..m}N_{c\left(i\right)}\frac{A_{\left(ij\right)}}{AF_{\left(i\right)}}}$ for vaccinated badgers and $\left(1-VC\right)\beta_{c,ub}S_{ub\left(j\right)}\frac{\sum_{i=1,\;..m}E_{c\left(i\right)}\frac{A_{\left(ij\right)}}{AF_{\left(i\right)}}}{\sum_{i=1..m}N_{c\left(i\right)}\frac{A_{\left(ij\right)}}{AF_{\left(i\right)}}}$ for unvaccinated badgers.

### 2.2. Model parameterisation

There are 14 parameters in this model. Six model parameters were estimated from the literature (Table 1). The details on the explanation and references for those parameters can be found in Supplementary Table 1. In addition, the transmission rate and decay rate parameters of *M. bovis* in the environment are estimated by fitting time-series infection data into a dose–response function (Section 2.3).

**Table 1:** Model parameters.

**Parameter**	**Description**	**Value**
β_*c, c*_	Transmission rate parameter from cattle to cattle	Estimated
β_*b, c*_	Transmission rate parameter from badges to cattle	Estimated
$\beta_{b,ub}\frac{N_{b}}{N_{c}}$	Transmission rate parameter from badger to unvaccinated badger	Estimated
$\beta_{c,ub}\frac{N_{b}}{N_{c}}$	Transmission rate parameter from cattle to unvaccinated badger	Estimated
$\beta_{b,vb}\frac{N_{b}}{N_{c}}$	Transmission rate parameter from badger to vaccinated badger	Estimated
$\beta_{c,vb}\frac{N_{b}}{N_{c}}$	Transmission rate parameter from cattle to vaccinated badger	Estimated
φ	The shedding rate parameter of *M. bovis*	Standardized
μ	*M. bovis* decay rate parameter	Estimated
$\frac{1}{\gamma_{c}}$	Infectious period for cattle	101 days
$\frac{1}{\gamma_{b}}$	Infectious period for badgers	365 days
$\frac{1}{\lambda_{c}}$	Latent period for cattle	1.8 days
$\frac{1}{\lambda_{b}}$	Latent period for badgers	90 days
α_*c*_	The cattle background death rate	9.13e-4 day^−1^
α_*b*_	The badger natural death rate	7.52e-4 day^−1^

### 2.3. Statistical analysis

We estimate transmission rate and decay rate parameters by fitting time-series infection data into the model. The core of this method is to relate exposure to hazards and the hazards to the infection probability (40). We first reconstruct the exposure in this two-host environmental transmission model (Section 2.3.1) and then fit the cattle and badger infection data and exposure to the statistical model to estimate transmission rate and decay rate parameters (Section 2.3.2).

#### 2.3.1. Reconstruction of exposure

From Eq. 1, we derive the environmental contamination (*E*(*t*)) as a function of time and the number of infectious individuals (Eq. 5). The exposure to the environmental contamination during one time interval is the integral of *E*(*t*) as $\int_{0}^{1}E\left(t|I_{t},E_{0}\right)\;$(Eq. 4).

(5) $$\begin{array}{l} E\left(t|I_{t},E_{0}\right)=\frac{\left(1{-}^{-t\mu }\right)\mu }{-1{+}^{-\mu }+\mu }I_{t}{+}^{-t\mu }E_{0} \end{array}$$

(6) $$\begin{array}{l} \int_{0}^{1}E\left(t|I_{t},E_{0}\right)\;=I_{t}\;+\frac{1{-}^{-\mu }}{\mu }E_{0}\; \end{array}$$

Here, *E*_0_ denotes the environmental contamination of at the start of an interval and *I*_*t*_ denotes the number of infectious individuals (cattle or badgers) during this interval. These equations were used to construct *E*_*c*_ and *E*_*b*_ and exposure by integrating each farm and territory.

#### 2.3.2. Likelihood function

The number of new cases over each observation time interval (τ, τ + Δ) follows a binomial distribution with a binomial total susceptible individual number at each time interval. The probability used in the binomial distribution is the probability of getting infected. From Eqs. 3, 4, the probability of getting infected can be derived for cattle and badgers, respectively, as follows:

(7) $$\begin{array}{l} P_{c}=1-e^{-}(\beta_{c,c}\frac{\int_{\tau }^{\tau +\Delta }E_{c\left(i\right)}\left(t|I_{c\left(i\right)\tau },E_{c\left(i\right)}\left(\tau \right)\right)dt}{N_{c\left(i\right)}}\\ +\beta_{b,c}\frac{\sum_{j=1..k}\left(\int_{\tau }^{\tau +\Delta }\left(E_{b\left(j\right)}{\left(t|I_{b\left(j\right)\tau },E_{b\left(j\right)}\left(\tau \right)\right)}^{*}\frac{A_{\left(ij\right)}}{AT_{\left(j\right)}}\right)dt\right)}{N_{c\left(i\right)}} \end{array}$$

(8) $$\begin{array}{l} P_{ub}=1-e^{-}(\beta_{b,ub}\frac{\int_{\tau }^{\tau +\Delta }E_{b\left(j\right)}\left(t|I_{b\left(j\right)\tau },E_{b\left(j\right)}\left(\tau \right)\right)dt}{\sum_{i=1..m}N_{c\left(i\right)}\frac{A_{\left(ij\right)}}{AF_{\left(i\right)}}}\\ +\beta_{c,ub}\frac{\sum_{i=1..m}\left(\int_{\tau }^{\tau +\Delta }\left(E_{c\left(i\right)}{\left(t|I_{c\left(i\right)\tau },E_{c\left(i\right)}\left(\tau \right)\right)}^{*}\frac{A_{\left(ij\right)}}{AF_{\left(i\right)}}\right)dt\right)}{\sum_{i=1..m}N_{c\left(i\right)}\frac{A_{\left(ij\right)}}{AF_{\left(i\right)}}}) \end{array}$$

(9) $$\begin{array}{l} P_{vb}=1-e^{-}(\beta_{b,vb}\frac{\int_{\tau }^{\tau +\Delta }E_{b\left(j\right)}\left(t|I_{b\left(j\right)\tau },E_{b\left(j\right)}\left(\tau \right)\right)dt}{\sum_{i=1..m}N_{c\left(i\right)}\frac{A_{\left(ij\right)}}{AF_{\left(i\right)}}}\\ +\beta_{c,vb}\frac{\sum_{i=1..m}\left(\int_{\tau }^{\tau +\Delta }\left(E_{c\left(i\right)}{\left(t|I_{c\left(i\right)\tau },E_{c\left(i\right)}\left(\tau \right)\right)}^{*}\frac{A_{\left(ij\right)}}{AF_{\left(i\right)}}\right)dt\right)}{\sum_{i=1..m}N_{c\left(i\right)}\frac{A_{\left(ij\right)}}{AF_{\left(i\right)}}}) \end{array}$$

where *I*_*c*(*i*)τ_, *I*_*ub*(*j*)τ_, and *I*_*vb*(*j*)τ_ represent the *I*_*c*_ at farm *I*, *I*_*ub*_, and *I*_*vb*_ at territory j at the beginning of (τ, τ+). *I*_*c*(*i*)τ_, *I*_*ub*(*j*)τ_, and *I*_*vb*(*j*)τ_ are integers and change discretely in jumps of 1. *E*_*c*(*i*)_(τ) and *E*_*b*(*j*)_(τ) represent *E*_*c*(*i*)_ and *E*_*b*(*j*)_ at time τ. *E*_*c*(*i*)_(τ) and *E*_*b*(*j*)_(τ) change continuously.

The likelihood as a function of transmission rate parameters and decay rate parameters is given by:

(10) $$\begin{array}{l} L\left(\theta \right)=\underset{x}{\prod }{\left(P\right)}^{cases_{x}}{\left(1-P\right)}^{\left(S_{x}-cases_{x}\right)} \end{array}$$

where *P* represents either *P*_*c*_, *P*_*ub*_, or *P*_*vb*_ from Eqs. 7–9.

### 2.4. Data

The infection data and geographic data for cattle and badgers are extracted to quantify parameters as described in Section 2.3. The new cases in each observation interval are used to calculate the probability of infection in each interval in Eqs. 7–9 and the prevalence at the beginning of each observation time interval in each spatial unit is used to reconstruct the exposure as described in Section 2.3.

#### 2.4.1. Badger data

The badger vaccination trial ran from 2009 to 2013 in the Kilkenny area (42). A 750 km^2^ study area was divided into three zones (A, B, and C) from north to south. Badger setts were identified and their locations recorded. Badgers were captured using cages or restraints. Blood samples were collected at each capture and tested using enzyme-linked immunosorbent assay (ELISA; (43)). Captured badgers were assigned to the sett closest to where they were trapped, with most captures taking place directly outside sett entrances. All the captured badgers in Zone A and 50% of the captured badgers in Zone B received a placebo. Half of the captured badgers in Zone B and all the captured badgers in Zone C received oral BCG vaccine (Danish strain 1331, at dose 10^8^ cfu).

Details of the badger infection dataset from the vaccination trial and the location of badger territories were described elsewhere (15, 44). In total, there were 1759 trapping records. Each record contains the information from the trapping of a single badger: badger ID, sett ID, infection status, date of examination, vaccine status, date of vaccination, vaccine code, etc. From all the trapping records, we extracted 440 pairs of trapping records from badgers that were captured more than once. Each pair of capture records consists of two examination results, namely the serology status at the beginning and the end of the interval, with the infectious status being negative at the beginning. Each pair of capture records has an outcome of 0 or 1 infection, which can be used to calculate the probability of infection during an interval, namely *P*_*ub*_ and *P*_*vb*_ in Eqs. 7–9.

In addition, the number of infectious badgers in each territory *j* at the time *x* (*I*_*b*(*j*)*x*_) is needed on the right side of Eqs. 7–9. We calculated *I*_*b*(*j*)*x*_ by multiplying the badger bTB prevalence by the number of badgers per territory. The number of badgers per territory was calculated using the minimum number alive. Badger prevalence was calculated from 1759 trapping results. The spatial and temporal resolution in the model is at territory and day levels, while the data are limited compared to the resolution in this model. Therefore, we fitted badger bTB prevalence at the territory level at different time points with several generalized additive models (GAMs) and then used the best-fitting GAM to predict the badger bTB prevalence for each day in each territory (see details about the GAMs in Supplementary Table 3). In addition, a sensitivity analysis was conducted to assess the impact of uncertainty in badger prevalence on the parameter estimation (see Supplementary Table 4).

#### 2.4.2. Cattle data

Cattle data were extracted from the Animal Health Computer System (AHCS) dataset and the Land Parcel Identification System (LPIS) of the Irish Government's Department of Agriculture, Food and the Marine (DAFM). The AHCS dataset comprises bTB test records on more than 98% of herds, including single intradermal comparative tuberculin test (SICTT), interferon-gamma array, ELISA test, and slaughterhouse inspection results. Herds are tested by the SICTT at least once a year. In this study, the sensitivity of tests was assumed to be 100%. Positive cattle are removed within 2–4 weeks of testing by staff from DAFM. In the AHCS dataset, each record consists of the number of cattle tested, the date of the test, the type of the test, the number of positive cattle, the number of inconclusive cattle, etc. When there are inconclusive tests in the herd, field veterinarians re-test the cattle or the herd within 3 months. From 2009 to 2013, there were 6787 test records from 1335 herds in this badger vaccination trial area. In all these events, 696 records from 390 herds were positive. In each data line, the number of new cases in a herd during an interval is the *P*_*c*_ in Eq. (7). The number of infected animals at the start of the interval time x (*I*_*c*(*i*)τ_) is used to construct the exposure (right side of Eq. 7).

The LPIS dataset delineates the land parcels making up each farm. Many Irish farms consist of several land fragments (45–47). For historic and topological reasons, the extent of fragmentation varies within Ireland. In the region of this study, approximately 20% of farms are single-fragment farms. The remaining 80% of farms have an average of five fragments, with a mean distance between same-farm fragments of 3.3 km. The movement within a herd but amongst different fragments was not recorded. Therefore, we assume that the time cattle spend on each fragment is proportional to the area of the fragment.

### 2.5. Basic reproduction ratio

#### 2.5.1. Within-herd R

The next-generation matrix (NGM) is a commonly used method to derive the basic reproduction ratio for a compartmental model (48). With the estimated transmission and decay rate parameters, we can calculate the basic reproduction ratio for this cattle badger system in a theoretical local area as: $\left[\begin{array}{c} R_{c,c},R_{b,c}\\ R_{c,b}\frac{N_{b}}{N_{c}},R_{b,b}\frac{N_{b}}{N_{c}} \end{array}\right]$, where

$$\begin{array}{ll} R_{c,c}=\;\frac{\beta_{c,c}\mu }{\left(-1+e^{-\mu }+\mu \right)}\frac{\lambda_{c}}{\left(\alpha_{c}+\lambda_{c}\right)}\frac{1}{\alpha_{c}+\;\gamma_{c}}\\ R_{b,c}=\;\frac{\beta_{c,c}\mu }{\left(-1+e^{-\mu }+\mu \right)}\frac{\lambda_{c}}{\left(\alpha_{c}+\lambda_{c}\right)}\frac{1}{\alpha_{b}+\;\gamma_{b}}\\ R_{c,b}=VC^{*}\left(\frac{\beta_{c,vb}\mu }{\left(-1+e^{-\mu }+\mu \right)}\frac{\lambda_{b}}{\left(\alpha_{b}+\lambda_{b}\right)}\frac{1}{\alpha_{c}+\gamma_{c}}\right)\\ + & {\left(1-VC\right)}^{*}\frac{\beta_{c,ub}\mu }{\left(-1+e^{-\mu }+\mu \right)}\frac{\lambda_{b}}{\left(\alpha_{b}+\lambda_{b}\right)}\frac{1}{\alpha_{c}+\gamma_{c}}\\ R_{b,b}=VC^{*}\frac{\beta_{b,vb}\mu }{\left(-1+e^{-\mu }+\mu \right)}\frac{\lambda_{b}}{\left(\alpha_{b}+\lambda_{b}\right)}\frac{1}{\alpha_{b}+\gamma_{b}}\\ + & {\left(1-VC\right)}^{*}\frac{\beta_{b,ub}\mu }{\left(-1+e^{-\mu }+\mu \right)}\frac{\lambda_{b}}{\left(\alpha_{b}+\lambda_{b}\right)}\frac{1}{\alpha_{b}+\gamma_{b}}. \end{array}$$

$\frac{N_{b}}{N_{c}}$ represents the relative badger density compared to cattle in a local area. We used this term rather than the term relative abundance because in our model, *N*_*c*_ is a proxy of the area under consideration, with the implicit assumption that cattle density is spatially uniform. Thus, the relative badger density cannot be reduced by simply increasing the number of cattle, as such an increase would mean an enlargement of the land area. *VC* represents the vaccination coverage, and (1 – *VC*) represents the proportion of unvaccinated badgers. We use *VC* = 0% and 100% to calculate the partial reproduction ratio in unvaccinated and fully vaccinated areas. The largest eigenvalue of this matrix is the basic reproduction ratio within this local area, which is derived as follows:

(11) $$\begin{array}{r} R=\frac{1}{2}\left(R_{c,c}\;+\;R_{b,b}\frac{N_{b}}{N_{c}}\;\right)\\ +\;\frac{1}{2}\sqrt{{\left(R_{c,c}\;+\;R_{b,b}\frac{N_{b}}{N_{c}}\right)}^{2}-4\left(R_{c,c}R_{b,b}\frac{N_{b}}{N_{c}}-\;R_{c,b}R_{b,c}\frac{N_{b}}{N_{c}}\right)} \end{array}$$

*R* represents the average number of new infections per case within this isolated local area, such as a farm with a badger territory lying completely inside the farm.

However, in reality, badgers' territories connect multiple local areas. Badgers act as vectors in the sense that they get infected by one herd and transmit infection to cattle in other herds. When an infectious bovine is introduced to a herd or an infectious badger comes into contact with a herd, there is a risk that infection will be spread to neighboring herds by badgers. To control bTB spread, we need to evaluate both within- and between-herd transmission.

#### 2.5.2. Between-herd R

The average number of neighboring herds infected by a single newly infected farm is denoted by the between-herd R. To calculate the between-herd R, a stochastic metapopulation model for each herd and its neighboring herds was developed with the same model structure as described in Figure 1 using the SimInf package in R (49). All the infection and vital dynamic processes are modeled stochastically using the Gillespie Algorithm, while *M. bovis* dynamic shedding and decay in the environment are modeled deterministically in Eqs. 1, 2. The spatial structure was accounted for according to Eqs. 3–4. In the Kilkenny area, there are a total of 1335 herds. For each herd, we simulated the transmission between the herd itself, the connected badger territories, and the herds that are directly connected (i.e. those that share a connected badger territory with the initial herd). In total, 1335 different spatial configurations were simulated, each with 200 repetitions.

Parameter estimations obtained in the analyses in Sections 2.2 and 2.3 were used in this simulation. In the initial state, one infectious bovine is introduced to a herd. Badgers are considered fully susceptible, and there is no contamination in the environment. The resulting distribution for the number of infected neighboring herds represents the between-herd R distribution. The average number of infected herds is the between-herd R.

## 3. Results

### 3.1. Parameter estimations

The decay rate parameter is estimated as 0.0039 day^−1^ with CI (0.0036, 0.0041), which means the half-life of *M. bovis* is 178 days, ranging from 169 to 192 days. Transmission rate parameters are estimated with a unit of per day for one infectious individual (Table 2). In addition, our parameter estimation is robust across the varying assumptions used to calculate badger prevalence (Supplementary Table 4).

**Table 2:** Parameter estimation.

**Parameter**	**Estimation (per day per E unit)**	**CI**	**Transformed value (per individual per year)**	**CI**
β_*c,c*_	1.01e-5	(9.7e-6, 1.07e-5)	1.89	(1.82, 1.97)
β_*b,c*_	3.977e-6	(3.78e-6, 4.19e-6)	0.756	(0.71, 0.78)
$\beta_{b,vb}\frac{N_{b}}{N_{c}}$	5.14e-5 $\frac{N_{b}}{N_{c}}$	(3.34e-5, 7.28e-5) $\frac{N_{b}}{N_{c}}$	9.63 $\frac{N_{b}}{N_{c}}$	(6.26, 13.64) $\frac{N_{b}}{N_{c}}$
$\beta_{c,vb}\frac{N_{b}}{N_{c}}$	4.43e-4 $\frac{N_{b}}{N_{c}}$	(2.64e-4, 6.62e-4) $\frac{N_{b}}{N_{c}}$	82.95 $\frac{N_{b}}{N_{c}}$	(49.62, 124.07) $\frac{N_{b}}{N_{c}}$
$\beta_{b,ub}\frac{N_{b}}{N_{c}}$	9.19e-5 $\frac{N_{b}}{N_{c}}$	(6.44e-5, 1.23e-4) $\frac{N_{b}}{N_{c}}$	17.22 $\frac{N_{b}}{N_{c}}$	(12.09, 23.21) $\frac{N_{b}}{N_{c}}$
$\beta_{c,ub}\frac{N_{b}}{N_{c}}$	5.07e-4 $\frac{N_{b}}{N_{c}}$	(2.98e-4, 7.62e-4) $\frac{N_{b}}{N_{c}}$	95.13 $\frac{N_{b}}{N_{c}}$	(55.83, 142.87) $\frac{N_{b}}{N_{c}}$

We transform β_*cc*_ to a yearly rate per infected individual (${\frac{\beta_{cc}\mu }{\left(-1+e^{-\mu }+\mu \right)}}^{*}365$) for comparison with other transmission models that use direct contact assumptions. One infectious bovine can infect on average 1.97 cattle per year in a fully susceptible herd with CI (1.82, 1.97). This estimation is slightly lower than estimations in New Zealand, the Netherlands, and Argentina, ranging from 2.2 to 5.2 per year (50–53).

The transmission rate parameter for badgers (β_*b,vb*_, β_*c,vb*_, β_*b,ub*_, and β_*c,vb*_) need to be interpreted with a multiplication of the local relative badger density (see NGM in Section 2.5), hence they cannot be directly compared with transmission rate parameters for cattle (β_*c,c*_, β_*b,c*_). For example, in an area with $\frac{N_{b}}{N_{c}}=0.01$, an infectious bovine can infect on average 0.95 unvaccinated badgers per year with CI (0.56, 1.42).

### 3.2. Within-herd R

In an isolated farm that does not connect to other farms, the within-herd R can be derived based on the methods presented in Section 2.5.1. When badgers are unvaccinated, the NGM for this farm is $\left[\begin{array}{c} 0.49,0.59\\ \;22.11\frac{N_{b}}{N_{c}},14.04\frac{N_{b}}{N_{c}} \end{array}\right]$, where $\frac{N_{b}}{N_{c}}$ represents the relative badger density in the farm. When badgers are vaccinated, the NGM is $\left[\begin{array}{c} 0.49,0.59\\ \;20.02\frac{N_{b}}{N_{c}},8.22\frac{N_{b}}{N_{c}} \end{array}\right]\;$. When an infectious bovine is introduced on this isolated farm, it will infect 0.49 cattle on average during its infectious period. In comparison, when an infectious badger is introduced, it will infect, on average, 0.59 cattle. The shorter infectious period of cattle than badgers leads to a smaller *R*_*cc*_ than *R*_*bc*_. However, a relaxation of the test-and-removal strategy will lead to a longer cattle infectious period and thus increase *R*_*cc*_.

**Figure 3:**
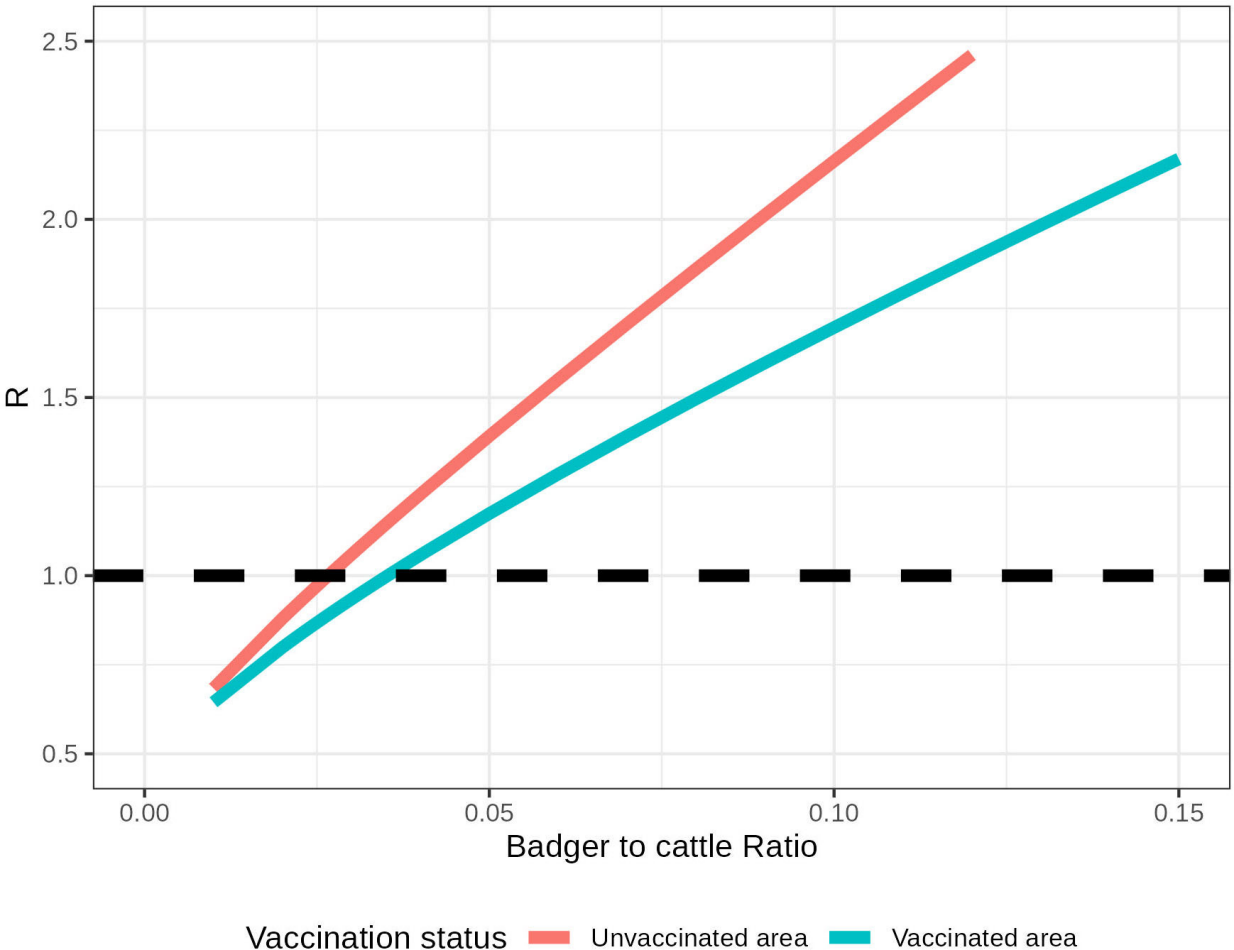
Within-herd R in an isolated herd with different relative badger densities (Nb/Nc). The pink line represents the within-herd R without badger vaccination, and the blue line represents the within-herd R with badger vaccination. The black dashed line represents the threshold R = 1.

The number of infected badgers in this system depends on the relative badger density ($\frac{N_{b}}{N_{c}}$). In addition, the impact of badger vaccination on within-herd R depends on the $\frac{N_{b}}{N_{c}}.$ For example, in a herd with 100 cattle and three unvaccinated badgers, the within-herd R for this local area is 1.08. If all badgers are vaccinated in this local area, the within-herd R is 0.97 (Figure 3). For example, to control R < 1 within an isolated area that accommodates 100 cattle, the relative badger density should be less than 2.5 unvaccinated badgers or 3.2 vaccinated badgers. As the relative badger density and the system R are highly correlated (with a correlation coefficient of 0.999), we fit them into a linear regression. In estimated linear relationships, R increases by 0.134 when the $\frac{N_{b}}{N_{c}}$ increases by 0.01 in an unvaccinated area. With all the badgers being vaccinated, this increase in R per 0.01 $\frac{N_{b}}{N_{c}}$ is reduced to 0.084.

### 3.3. Between-herd R

In real life, herds are not isolated but connected with each other by badger territories. Even if each isolated area has an R below 1, bTB might still spread from one local area to another. Therefore, we used simulations to calculate the average number of herds that get infected if an infectious bovine is introduced or tested positive in an index herd.

**Figure 4:**
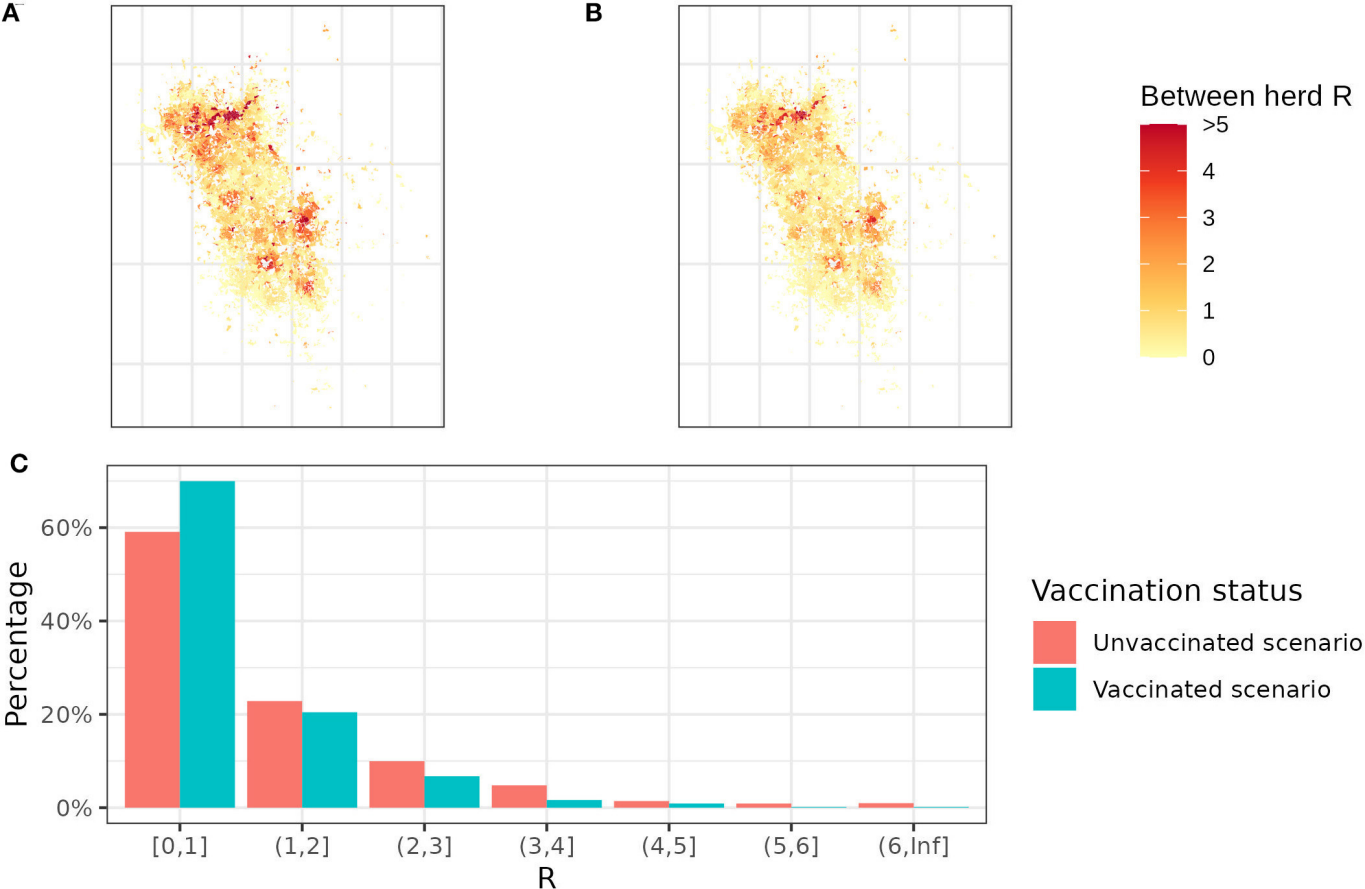
Between-herd R maps and R distribution. **(A)** The between-herd R map without any badger vaccination and **(B)** the between-herd R map with 100% vaccination coverage. Yellow herds represent between-herd R below 1, while orange and red herds represent between-herd R >1. **(C)** The distribution of between-herd R with and without badger vaccination. Each bar represents the percentage of herds falling within a specific between-herd R range. For example, the first bar indicates that 70% of herds have between-herd R < 1 in the vaccination scenario and 60% in the un-vaccination scenario.

In between-herd R maps (Figure 4), herds in yellow are expected to spread bTB to fewer than 1 neighboring herd, while herds in orange and red are expected to spread to more than 1 neighboring herd. Red areas are mostly clustered on the north and east sides of the study area due to the higher density of badgers. Some sporadic red dots lie in the yellow area because of the farm fragmentation, where high R herds have some land parcels in the low R herd clusters. By comparing the two maps, vaccination reduces the average between-herd R from 1.21 to 0.85. It is worth noting that the average between-herd R is being used to allow a quantitative comparison between maps but does not infer the bTB persistence in a whole area. Despite a 10% decrease in herds with R >1, there are still 30% of herds that can transmit bTB to more than 1 herd with the badger vaccination (Figure 4).

## 4. Discussion

The quantification of bTB transmission between wildlife and cattle is critical for efforts to eradicate bTB. In Ireland and the UK, recent studies have provided evidence that badgers are involved in maintaining bTB transmission; however, a quantitative understanding of how relative badger density influences transmission in this cattle and badger episystem has so far been lacking. To address this gap, this study quantifies the role of badgers, cattle, and the environment in bTB transmission and disentangles how relative badger density may contribute to the spatial heterogeneity in bTB transmission. To achieve this objective, we developed a novel environmental transmission model that incorporates both within-herd/badger territory transmission and between-species transmission. This approach is guided by the overlap of badger territories with cattle herds.

In this two-host transmission system, the partial reproduction ratio *R*_*bc*_ is higher than *R*_*cc*_. This is because badgers likely remain infectious for a longer period than cattle, given the test and removal policy in cattle in place. Therefore, any relaxation of the test-and-removal policy can lead to higher *R*_*cc*_. The partial reproduction ratios $R_{c,\left(u/v\right)b}\frac{N_{b}}{N_{c}}$ and $R_{b,\left(u/v\right)b}\frac{N_{b}}{N_{c}}$ depend on the local relative badger density ($\frac{N_{b}}{N_{c}}$). As a result, we quantified the relationship between local relative badger density and the R for the system. In unvaccinated areas, within-herd R increases by 0.134 for every 0.01 increase in the $\frac{N_{b}}{N_{c}}$. This increase is reduced to 0.084 per 0.01 increase in $\frac{N_{b}}{N_{c}}$ when badgers are vaccinated.

Our transmission model adopts a single transmission route, incorporating an environmental compartment. We simplified droplet, aerosol, fecal to oral transmissions to one environmental transmission route as they are intrinsically similar. The primary distinction lies in the duration between the shedding moment and the time point of inhaling or ingesting *M. bovis*. Shortly after being shed into the environment, *M. bovis* cells may pose an infection risk to other animals. This infection risk decreases over time because viable *M. bovis* decays over time in the environment. We unified these three transmission routes into one and assumed an exponential decay of *M. bovis* with a specified decay rate. This unification simplifies the model structure while still capturing the significance of historic infections. In addition, badger-to-badger transmission via biting may represent a secondary route of infection, which has not been considered in this study. Previous studies have shown that transmission via biting can cause a more rapid and progressive infection with generalized pathology (54). The simplification of transmission routes might lead to an underestimation of badger-to-badger transmission and an overestimation of cattle-to-badger transmission. However, it is not our goal to distinguish badger infection via biting or the other three mechanisms, as the data to distinguish the contribution of different mechanisms are lacking.

Previous studies on the within-herd transmission of bTB have exploited either frequency- or density-dependent models (50, 52, 55). A study in US dairy herds found that a frequency-dependent model can predict risk significantly better than a density-dependent model (55). Additionally, Conlan et al. (56) measured the strength of the density dependence of transmission and found a non-linear dependence with herd size. Therefore, our model adopts a frequency-dependent model and uses the number of cattle as a proxy for the area in transmission rates (Eqs. 3, 4). This approximation is valid in areas where badger territories and farms dominate a significant portion of the region frequented by badgers, as in this study area. However, when a significant portion of the region consists of woodlands, rivers, and urban areas, it becomes crucial to modify this proxy. This adjustment is necessary to avoid underestimating the denominator in the badger-to-badger transmission rate, which could otherwise result in an overestimation of the badger-to-badger transmission rate parameter. In addition to using cattle numbers as a proxy for area, one can consider alternative denominators such as the number of badgers or the sum of cattle and badgers. Our assessment showed that models with Nc or Nc + Nb as the denominator in the transmission rates (in Eqs. 3, 4) provided similar results in fitting the data (see Supplementary Table 5).

The significance of the environment in the transmission of *M. bovis* is emphasized in our model, which estimates a half-life of 6 months. Our estimation of the half-life of *M. bovis* in the environment is five times higher in comparison to other modeling studies (39), although still within the range of experimental studies (31, 57). We also conducted a sensitivity analysis of the decay rate using the estimates from (39). A shorter survival time of *M. bovis* can lead to an increase in transmission rate parameters, but the outcome of this study with respect to NGM, R, and the threshold for relative badger density remain largely unaffected (see Supplementary Table 6).

The parameters defining the duration of intermediate stages of the disease (latent periods) were derived from the literature (see Supplementary Table 1). We did not estimate them from infection data because previous modeling studies have not been able to distinguish models with differing assumptions regarding these intermediate stages (SORI or SOR model) based on model fit (56). The most debated parameter is the latent period for cattle. Conventionally, it is believed that *M. bovis* can cause a long latent period similar to human TB. However, an animal challenge study showed that acute infection may occur (58). In addition, a recent review also suggests that *M. bovis* can frequently cause acute infection in cattle (59). Therefore, we also assume a short latent period for cattle. In this model, assuming a different latent period for cattle or badgers would impact the transmission rate parameter estimates. However, such a variation would not influence the values for R and NGM since the modifications to these β and λ would counterbalance each other within the R formula as described in Section 2.5.1. In addition, the sensitivity of tests for cattle and badgers is assumed to be perfect in this model. Infected but undetected animals shed *M. bovis*, which causes an underestimation of environmental contamination. On the other hand, these hidden infections cause an underestimation of the new cases. Both the left and right sides of Eqs. 7–9 were underestimated, whose effects are likely to be canceled out and therefore have a limited impact on the transmission rate parameter.

In this model, cattle and badgers are assumed to spend their time homogenously distributed within their spatial units. This is a simplification of reality, as some parcels of farms might not be used for grazing, or not all of the time, and badgers may spend more time near setts than elsewhere in their territories. However, as cattle and badger numbers and infection data are available at the farm and territory level, we used this as the spatial resolution for our model. Within-farm and within-territory heterogeneity might lead to an underestimation of the actual densities at the location of an infected animal, which in turn leads to an underestimation of the within-herd R by the model. However, heterogeneity in densities may also lead to less overlap in areas used by cattle and badgers, which would have the opposite effect. In addition, the assumption that animals are restricted to their spatial units, might attribute movement-mediated transmission to between-species transmission in the model. This can result in an overestimation of the between-species transmission. Future studies could relax this assumption and capture the effect of cattle movements using detailed cattle movement data.

In conclusion, this model disentangles the quantitative relationship between relative badger density and local transmission risks. Estimating transmission rate parameters improves our understanding of badgers as a vector in this two-host system. In addition, the model produces the first between-herd R map for bTB considering badger, cattle, and environment. These R maps identify high-risk areas as clusters of farms with between-herd R >1 and demonstrate how relative badger density determines the local transmission risk. Our results suggest that badger vaccination can maximally reduce the average between-herd R in Kilkenny to 0.85; however, despite this, 30% of herds will still have an R value >1 and so, if infected, have a high potential risk of transmitting bTB to their neighbors. Whether these 30% of herds with a high between-herd R can sustain the bTB spread in a large area, such as the whole Kilkenny area, is unknown and requires further research.

## Data availability statement

The datasets presented in this study can be found in online repositories. The names of the repository/repositories and accession number(s) can be found at: GitHub; https://git.wur.nl/chang025/btb_transmission_and_r_map.

## Author contributions

YC, NH, and MdJ contributed to the conception and design of the study. YC performed the statistical analysis, interpreted the results, and drafted the manuscript. NH and MdJ participated in the data analysis and manuscript preparation. AB, EG, GM, and JT organized and provided datasets for this study. PB, SM, AB, EG, GM, and JT critically revised the manuscript. All authors contributed to the manuscript revision and approved the submitted version.
