# Supplementary material for: Inferring bovine tuberculosis transmission between cattle and badgers via the environment and risk mapping
Source: Front Vet Sci. 2023 Sep 22;10:1233173. doi: 10.3389/fvets.2023.1233173 (PMC10572351; doi:10.3389/fvets.2023.1233173)
Supplement: Supplementary file 1 [file Presentation_1.pdf]

Supplementary Material

Inferring Bovine Tuberculosis Transmission Between Cattle and Badger via Environment and Risk Mapping

You Chang*, Nienke Hartemink, Andrew W. Byrne, Eamonn Gormley, Guy McGrath, Jamie A. Tratalos, Philip Breslin, Simon J. More, Mart C.M. de Jong

*** Correspondence:** You Chang: you.chang@wur.nl

1 Model Parameterization

We describe the details of infectious period, latent period and natural death rate for cattle and badgers in this section (Table 2).

Supplementary Table 1. Model parameterization

Parameter	Description	Value	Range	Source
$1/\gamma_c$	Infectious period for cattle	101 days	(30, 225) days	AHCS data
$1/\gamma_b$	Infectious period for badger	365 days	(117, 1305) days	(Little et al., 1982; Anderson & Trewhella, 1985; Gallagher et al., 1998)
$1/\lambda_c$	Latent period for cattle	1.8 days	(1.5, 600) days	(Barlow et al., 1997; Fischer et al., 2005; Conlan et al., 2012)
$1/\lambda_b$	Latent period for badger	90 days	(90, 158) days	(Anderson & Trewhella, 1985; Gallagher et al., 1998)

α_c	The cattle background death rate	1/3 year^{-1}	(M. H. Poola, 2005; Maher et al., 2008)
α_b	The badger natural death rate	1/1330 day^{-1}	(Anderson & Trewhella, 1985; Rogers et al., 1997)

1.1 Infectious period

The infectious period of cattle in ROI depends on the test frequency. The onset of the infectious period of a bovine is usually not exactly known. Therefore, we assume that cattle get infected at the middle point of two tests. We extract cattle testing data results in Kilkenny trial area during the vaccination study and calculate the average duration infectious period. Details on test scheme are explained below in 2.4.2 cattle data.

Once badgers get infected, they seem to have a life-long infectiousness. However, the excretion may be intermittent and vary greatly throughout time. The effective duration of infection period can be approximately derived as the average life span of infected animals (excluding the latent period) (Anderson & Trewhella, 1985). The life expectancy after bTB infection varies from 35 days to 3.5 years in in laboratory studies, with the most of badgers survive between one to two years (Little et al., 1982; Cheeseman et al., 1985). Another study also found that badgers with bite infection usually have more acute disease progress and the survival of those badgers with bite infection was estimated as 117 days (CI 0 to 341 days) (Clifton-Hadley et al., 1993). However, badgers who have apparent respiratory origin infection have a mean survival time of 491 days with CI 253 to 729 days (Clifton-Hadley et al., 1993).Based on all those information, we assume a one year infection period for badgers.

1.2 Latent period

The latent period of bTB in badgers is not well understood due to a lack of effective method of detecting *M. bovis* in live animals. Culture and examination methods have been used in some old experimental studies. However, those methods are labour intensive and prone to error, and should be interpreted with caution (Anderson & Trewhella, 1985). Little et al. (1982) undertook an animal experiment with a small sample size and used the duration between the first exposure to the first time of recording *M. bovis* excretion. This study found the range of latent period is from 95 days to 158 days, with a comment that the excretion is intermittent. Since the previous badger transmission model assumed a 3-month latent period based on those estimation, we used the same assumption (Anderson & Trewhella, 1985).

In cattle, animal challenge study has found a short latent period ~30 days (Kao et al., 2007), while other models have assumed a lengthy latent period of 6 to 20 months (Barlow et al., 1997; Fischer et al., 2005). A more recent modelling study compared two models with long or short latency but could not distinguish the two assumption (SOR and SORI) from within herd transmission data (Conlan et al., 2012). In SOR model, cattle are infectious once infected, but cattle cannot be detected in occult stage. In this assumption, occult cattle are estimated to become responsive to test cattle in 1.8 days. In the other assumption, infected cattle go through occult, responsive to test and then infectious. In this assumption, cattle's latent period is estimated to be 406 days or 28 days based on different prior

information with the similar model fit. As the animal experiments also suggest short latent period, we adopt a short latent period model assuming the latent period as 1.8 days (Conlan et al., 2012).

1.3 Background death rate

The natural death rate for badgers is calculated from the capture-mark-recapture study conducted in an undisturbed wild population in the west of England (Rogers et al., 1997). A survival probability each year was reported in the study. We assume an exponential distribution of death and therefore can derive an average lifespan of 1330 days with a natural death rate of 7.52e-4 per day.

The lifespan of cattle varies between farms, herd types and so on. In the Irish farming system, the average lifespan for beef breeds is almost 3 years, being slightly lower than for dairy breeds (M. H. Poola, 2005). Another study suggests that the annual culling rate is about 20% (Maher et al., 2008). Therefore, we assume the background death rate for cattle is about 1/3 per year (9.13e-4 per day).

2 Confidence bounds for partial reproduction ratio

We calculate the confidence bounds for partial reproduction ratios based on the confidence bounds of the transmission rates and the decay rate parameters estimation. The R_{cc} and R_{bc} have narrow confidence bounds than other partial R because R_{cc} and R_{bc} are estimated from the cattle infection data, which are more abundant than badger infection data.

Supplementary Table 2 Confidence bound for partial reproduction ratios

$R_{c,c}$	0.49	(0.46, 0.50)
$R_{b,c}$	0.58	(0.55, 0.61)
$R_{b,vb}\frac{N_b}{N_c}$	$7.07\frac{N_b}{N_c}$	$(4.60, 10.03)\frac{N_b}{N_c}$
$R_{c,vb}\frac{N_b}{N_c}$	$19.68\frac{N_b}{N_c}$	$(11.78, 29.44)\frac{N_b}{N_c}$
$R_{b,ub}\frac{N_b}{N_c}$	$12.6\frac{N_b}{N_c}$	$(8.88, 17.06)\frac{N_b}{N_c}$
$R_{c,ub}\frac{N_b}{N_c}$	$22.57\frac{N_b}{N_c}$	$(13.24, 33.90)\frac{N_b}{N_c}$

3 Smoothing badger prevalence data

Badger infection data were extracted from the badger vaccination trial in Kilkenny. Despite of the intensive data collection, the badger prevalence data are scarce at the resolution in this spatial model. Therefore, we use statistical learning to investigate the association between the spatial location of

badger territories, the time and the badger prevalence. With the best-fitted relationship, we can predict the badger in a finer resolution.

Badger annual prevalence at territory level has been calculated and fitted into several spline smooth models. The badger annual prevalence is the response of the model. The coordinates of the centroid of badger territories (x,y) and the time (day) are the three predictors in statistical model. The predictor (prevalence) has the value from 0 to 1, hence a binomial distribution with a logit link relationship between predictor and response is assumed.

We fit predictors to the observed prevalence data using smoothing spines. The goal was to find a function s() that fits the observed data well, while not overfitting the data. This means to find a function that minimizes $\sum_{i=1}^{n}(y_i - s(x_i))^2 + \lambda \int s''(t)^2 dt$, where λ is a tunning parameter and the function s() is the smoothing spline.

The functions for different predictors are additive, hence this statistical learning method is also called generalised additive models (GAM).Five statistical models with different predictors were tested:

Model 1: only time

$prevalences = s(t)$

Model 2: time and the vaccination zone

$prevalence = s(t) + s(zone)$

Model 3: coordinates (x,y)

$prevalence = s(x, y)$

Model 4: coordinates (x,y) and time

$prevalence = s(x, y) + s(t)$

Model 5: coordinates (x,y) and time with interaction

$prevalence = s(x, y, t)$

From the AIC of these 5 models, the best fit smoothing spline is the Model 5 where the coordinates (x, y) and t are the three predictors with interaction between coordinates and time (x*y*t);(Table 3).

Supplementary Table 3 AICfor smoothing models

Model	df	AIC
s(t)	2.0002	1771.910
s(t)+ s(zone)	4.0004	1776.133
s(x,y)	14.0123	1745.088
s(x,y) +s(t)	14.9310	1745.659
s(x,y,t)	72.4936	1723.466

The predicted prevalence over space is visualized in Figure 8. The predicted prevalence at each territory varies from 0 to 0.7 with the mean prevalence 0.28 (Figure 1B). Temporal prevalence changes in a few example badger territories are presented in Figure 9.

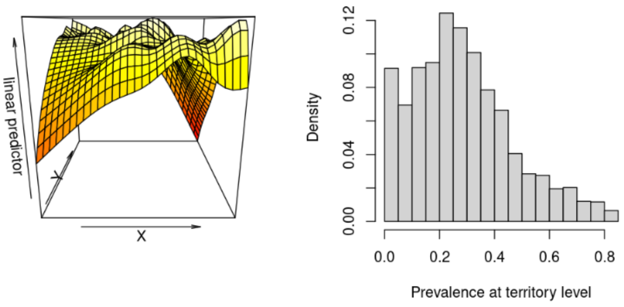

Supplementary Figure 2. The predicted badger prevalence by GAM s(x,y,t) model and the histogram of predicted prevalence at territory level.

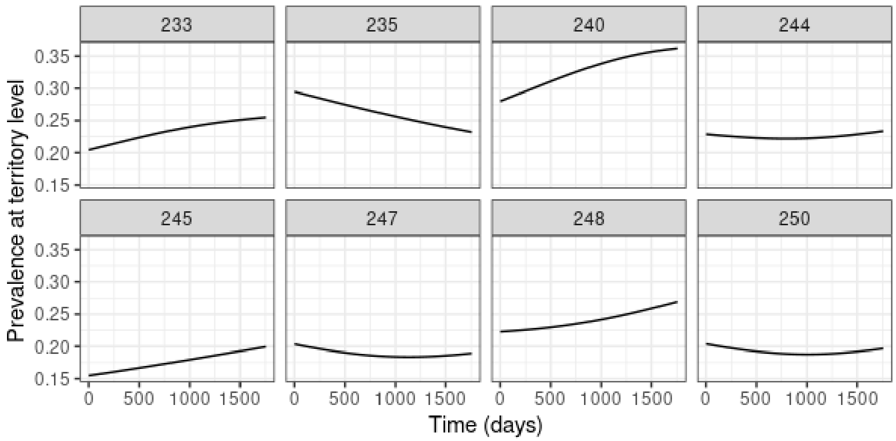

Supplementary Figure 2. An example of the predicted badger prevalence over time at some territories with the value on each panel representing the territory ID.

4 Impact of badger prevalence uncertainty on parameter estimation

Due to the scarce of badger data, the badger prevalence was estimated by GAM model might have uncertainty, which might cause some uncertainty. Therefore, we did sensitivity analysis on the badger prevalence by using two extreme scenarios: with a course spatial scale (at zone level) and fine temporal scale (day) or the other way around (territory level and no temporal pattern in 3 years). In two extreme scenarios, transmission rate parameter for cattle do not change at all, but transmission rate parameter to badger can be influenced. When calculating the badger prevalence at zone level, the transmission rate parameter are estimated to be higher.

Supplementary Table 4. Impact of badger prevalence on parameter estimation

	Territory level		Zone level	
Parameters	Estimation	CI	Estimation	CI
$\beta_{c,c}$	1.00E-05	(9.7e-6, 1.07e-5)	1.00E-05	(9.7e-6, 1.07e-5)
$\beta_{b,c}$	3.40E-06	(3.25e-6, 3.6e-6)	3.40E-06	(3.3e-6, 3.6e-6)
$\beta_{b,ub}$	3.00E-05	(2.4e-5, 3.8e-5)	4.10E-05	(2.5e-5, 6e-5)
$\beta_{c,ub}$	2.50E-05	(3e-6, 5.3e-5)	4.80E-04	(3e-4, 6.9e-4)
$\beta_{b,vb}$	2.00E-05	(1.4e-5, 2.8e-5)	6.10E-05	(4.2e-5, 8.4e-5)
$\beta_{c.vb}$	1.00E-04	(5.2e-5, 1.5e-4)	5.24E-04	(3.3e-4, 7.6e-4)

5 Model selection on different denominator in infection force

In this paper, we use the total number of cattle to represent a local. Badgers act as vectors that lives in farms but does not determine the area. The model structure is similar to vector-borne disease where the number of the host in an area is the denominator (Hartemink et al., 2009; Cecilia et al., 2020). In our case, the infection force is $\beta \frac{Exposure*S}{N_c}$ for both cattle and badgers. However, ones can assume different assumptions, such as using the number of badger or the cattle and badger number as the representation of this local area, with infection force as $\beta \frac{Exposure*S}{N_b}$ or $\beta \frac{Exposure*S}{N_c+N_b}$ under these two assumption respectively.

We select the three model structures by the goodness of fit. The model 3 (Nb+Nc) as denominator is the best fit model structure.

5.1 Nc determines the area

The ordinary differential equations (ODE) version of the transmission model can be written as:

$$\frac{dI_{c(i)}}{dt} = \beta_{cc} S_{c(i)} \frac{E_{c(i)}}{N_{c(i)}} + \beta_{bc} S_{c(i)} \frac{\sum_{j=1,..k} E_{b(j)} \frac{A_{(ij)}}{AT_{(j)}}}{N_{c(i)}}$$

$$\frac{dI_{ub(j)}}{dt} = \beta_{bub} S_{ub(j)} \frac{E_{ub(j)}}{\sum_{i=1..m} N_{c(i)} \frac{A_{(ij)}}{AF_{(i)}}} + \beta_{cub} S_{ub(j)} \frac{\sum_{i=1,..m} E_{c(i)} \frac{A_{(ij)}}{AF_{(i)}}}{\sum_{i=1..m} N_{c(i)} \frac{A_{(ij)}}{AF_{(i)}}} - \alpha_b I_{ub(j)}$$

$$\frac{dI_{vb(j)}}{dt} = \beta_{bvb} S_{vb(j)} \frac{E_{vb(j)}}{\sum_{i=1..m} N_{c(i)} \frac{A_{(ij)}}{AF_{(i)}}} + \beta_{cvb} S_{vb(j)} \frac{\sum_{i=1,..m} E_{c(i)} \frac{A_{(ij)}}{AF_{(i)}}}{\sum_{i=1..m} N_{c(i)} \frac{A_{(ij)}}{AF_{(i)}}} - \alpha_b I_{b(j)}$$

The next generation matrix of this model is:

$$\begin{bmatrix} R_{c,c} & R_{ub,c} & R_{vb,c} \\ R_{c,ub} \frac{N_b}{N_c} & R_{ub,ub} \frac{N_b}{N_c} & R_{vb,ub} \frac{N_b}{N_c} \\ R_{c,vb} \frac{N_b}{N_c} & R_{ub,vb} \frac{N_b}{N_c} & R_{vb,vb} \frac{N_b}{N_c} \end{bmatrix}$$

5.2: Nb determines the area

Compared to Model 1, all the denominator in ODE is changed to Nb.

$$\frac{dI_{c(i)}}{dt} = \beta_{cc} S_{c(i)} \frac{E_{c(i)}}{\sum_{j=1..n} N_{b(j)} \frac{A_{(ij)}}{AT_{(j)}}} + \beta_{bc} S_{c(i)} \frac{\sum_{j=1,..k} E_{b(j)} \frac{A_{(ij)}}{AT_{(j)}}}{\sum_{j=1..n} N_{b(j)} \frac{A_{(ij)}}{AT_{(j)}}}$$

$$\frac{dI_{ub(j)}}{dt} = \beta_{bub} S_{ub(j)} \frac{E_{ub(j)}}{N_{b(i)}} + \beta_{cub} S_{ub(j)} \frac{\sum_{i=1,..m} E_{c(i)} \frac{A_{(ij)}}{AF_{(i)}}}{N_{b(i)}} - \alpha_b I_{ub(j)}$$

$$\frac{dI_{vb(j)}}{dt} = \beta_{bvb} S_{vb(j)} \frac{E_{vb(j)}}{N_{b(i)}} + \beta_{cvb} S_{vb(j)} \frac{\sum_{i=1,..m} E_{c(i)} \frac{A_{(ij)}}{AF_{(i)}}}{N_{b(i)}} - \alpha_b I_{b(j)}$$

The next generation of this model is:

$$\begin{bmatrix} R_{c,c} \frac{N_c}{N_b} & R_{ub,c} \frac{N_c}{N_b} & R_{vb,c} \frac{N_c}{N_b} \\ R_{c,ub} & R_{ub,ub} & R_{vb,ub} \\ R_{c,vb} & R_{ub,vb} & R_{vb,vb} \end{bmatrix}$$

5.3: Nb +Nc determines the area

In the ODE version of transmission model, the denominator is the total number of cattle and badgers.

$$\frac{dI_{c(i)}}{dt} = \beta_{cc} S_{c(i)} \frac{E_{c(i)}}{\sum_{j=1..n} N_{b(j)} \frac{A_{(ij)}}{AT_{(j)}} + N_{c(i)}} + \beta_{bc} S_{c(i)} \frac{\sum_{j=1,..k} E_{b(j)} \frac{A_{(ij)}}{AT_{(j)}}}{\sum_{j=1..n} N_{b(j)} \frac{A_{(ij)}}{AT_{(j)}} + N_{c(i)}}$$

$$\frac{dI_{ub(j)}}{dt} = \beta_{bub} S_{ub(j)} \frac{E_{ub(j)}}{N_{b(i)} + \sum_{i=1..m} N_{c(i)} \frac{A_{(ij)}}{AF_{(i)}}} + \beta_{cub} S_{ub(j)} \frac{\sum_{i=1,..m} E_{c(i)} \frac{A_{(ij)}}{AF_{(i)}}}{N_{b(i)} + \sum_{i=1..m} N_{c(i)} \frac{A_{(ij)}}{AF_{(i)}}} - \alpha_b I_{ub(j)}$$

$$\frac{dI_{vb(j)}}{dt} = \beta_{bvb} S_{vb(j)} \frac{E_{vb(j)}}{N_{b(i)} + \sum_{i=1..m} N_{c(i)} \frac{A_{(ij)}}{AF_{(i)}}} + \beta_{cvb} S_{vb(j)} \frac{\sum_{i=1,..m} E_{c(i)} \frac{A_{(ij)}}{AF_{(i)}}}{N_{b(i)} + \sum_{i=1..m} N_{c(i)} \frac{A_{(ij)}}{AF_{(i)}}} - \alpha_b I_{b(j)}$$

The next generation matrix for this model is:

$$\begin{bmatrix} R_{c,c} \frac{N_c}{N_b + N_c} & R_{ub,c} \frac{N_c}{N_b + N_c} & R_{vb,c} \frac{N_c}{N_b + N_c} \\ R_{c,ub} \frac{N_b}{N_b + N_c} & R_{ub,ub} \frac{N_b}{N_b + N_c} & R_{vb,ub} \frac{N_b}{N_b + N_c} \\ R_{c,vb} \frac{N_b}{N_b + N_c} & R_{ub,vb} \frac{N_b}{N_b + N_c} & R_{vb,vb} \frac{N_b}{N_b + N_c} \end{bmatrix}$$

The statistic model descripted in 2.3 is used to fit these three models to estimate the parameters. The goodness of fit for these three models was used to select the best fit model (Table 4)

Supplementary Table 5. Goodness of fit for three transmission model structures

(denominator)	Data	Parameters	Estimation	AIC
Nc	Cattle	β_{cc}	1.05E-05	
		$\beta_{b,c}$	3.98E-06	15925.2
		$\beta_{b,ub}$	9.19E-05	301.4

	Unvaccinated badgers	$\beta_{c,ub}$	5.07E-04	
	Vaccinated badgers	$\beta_{b,vb}$	5.14E-05	
		$\beta_{c.vb}$	4.43E-04	253.9
	Total AIC			16480.5
Nb	Cattle	β_{cc}	8.63E-09	
		$\beta_{b,c}$	5.77E-08	16142.8
	Unvaccinated badger badgers	$\beta_{b,ub}$	5.22E-06	
		$\beta_{c,ub}$	1.12E-06	261.9
	Vaccinated badgers	$\beta_{b,vb}$	3.37E-06	
		$\beta_{c.vb}$	2.17E-06	224.4
	Total AIC			16629.1
Nc+Nb	Cattle	β_{cc}	1.04E-05	15896.39
		$\beta_{b,c}$	4.07E-06	
	Unvaccinated badger badgers	$\beta_{b,ub}$	9.55E-05	298.7
		$\beta_{c,ub}$	4.66E-04	
	Vaccinated badgers	$\beta_{b,vb}$	5.59E-05	249.6
		$\beta_{c.vb}$	4.22E-04	
	Total AIC			16444.69

6 Sensitivity analysis of decay rate

M.bovis's half -life is estimated to 35 days ($\mu = 0.02$) from Brooks-Pollock and Wood (2015), which is 5 times higher than the estimation in this study (half-life 177 days; $\mu = 0.004$). Therefore, we conduct a sensitivity analysis to investigate how would decay rate parameter influence the result of this study in terms of estimation on transmission rate parameter, R, and badger-cattle ratio threshold.

Supplementary Table 6. Sensitivity test of decay rate on transmission rate parameter estimation

Data	Parameters	$\mu = 0.004$	$\mu = 0.02$
		Estimation	Estimation
Cattle	β_{cc}	1.03E-05	3.56E-5
	$\beta_{b,c}$	3.97E-06	2.51E-5
Unvaccinated badgers	$\beta_{b,ub}$	9.19E-05	5.3E-4
	$\beta_{c,ub}$	5.07E-04	1.7E-3
Vaccinated badgers	$\beta_{b,vb}$	5.14E-05	3.2E-4
	$\beta_{c.vb}$	4.43E-04	1.6E-3
AIC		16480.5	17531.46

Despite the changes in transmission rate parameters, the decay rate parameter has limited impact on the partial R value. From the point estimation, higher decay rate (μ =0.02) resulted in higher $R_{b,c}$ and $R_{b,b}$. However, the changes in the partial R does not influence much on the R for the system (Figure 10).

Supplementary Table 7. Sensitivity test of decay rate on NGM

	$\mu = 0.004$	$\mu = 0.02$
No vac	$\begin{bmatrix} 0.49 & 0.59 \\ 22.11\frac{N_b}{N_c} & 14.04\frac{N_b}{N_c} \end{bmatrix}$	$\begin{bmatrix} 0.3 & 0.80 \\ 15.28\frac{N_b}{N_c} & 15.83\frac{N_b}{N_c} \end{bmatrix}$

Vac	$\begin{bmatrix} 0.49 & 0.59 \\ 20.02\frac{N_b}{N_c} & 8.22\frac{N_b}{N_c} \end{bmatrix}$	$\begin{bmatrix} 0.3 & 0.80 \\ 14.89\frac{N_b}{N_c} & 9.22\frac{N_b}{N_c} \end{bmatrix}$

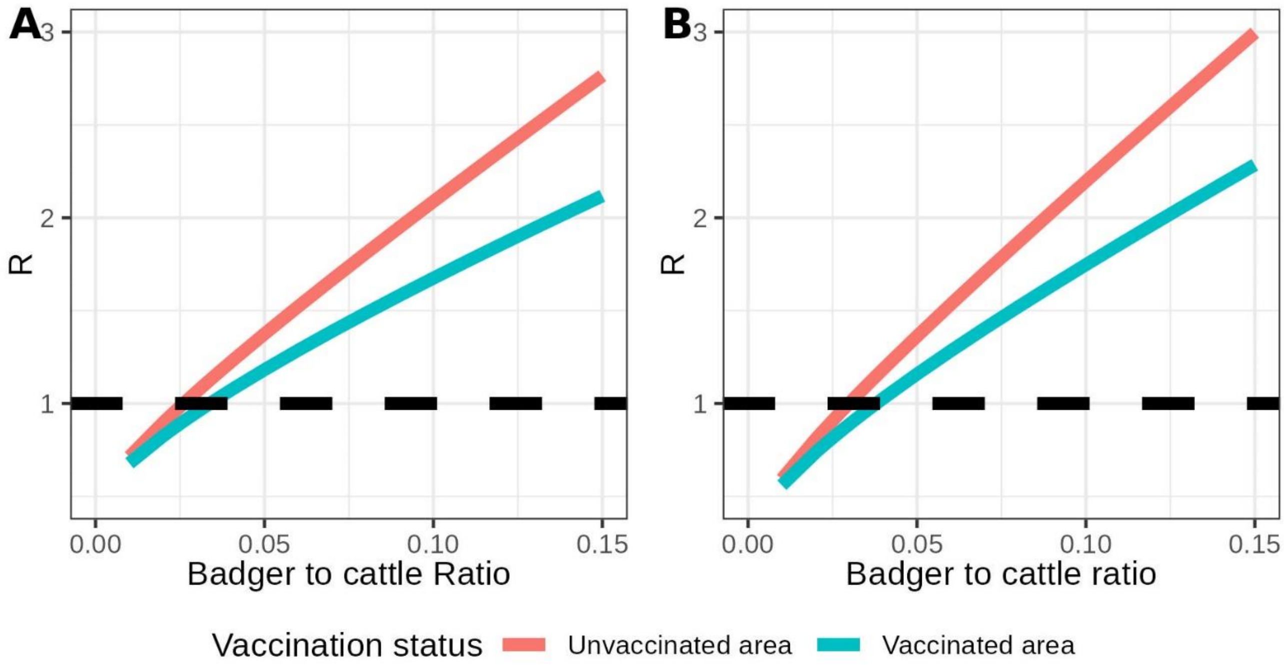

Supplementary Figure 3. Within-herd R in an isolated herd with different relative badger density. A)$\boldsymbol{\mu = 0.004}$; B) $\boldsymbol{\mu = 0.02}$

7 Reference

Anderson, R. M., & Trewhella, W. (1985). Population dynamics of the badger (Meles meles) and the epidemiology of bovine tuberculosis (Mycobacterium bovis). Philosophical Transactions of the Royal Society of London. B, Biological Sciences, 310(1145), 327-381.

Barlow, N. D., Kean, J. M., Hickling, G., Livingstone, P. G., & Robson, A. B. (1997). A simulation model for the spread of bovine tuberculosis within New Zealand cattle herds. Preventive veterinary medicine, 32(1-2), 57-75.

Brooks-Pollock, E., & Wood, J. L. (2015). Eliminating bovine tuberculosis in cattle and badgers: insight from a dynamic model. Proc Biol Sci, 282(1808), 20150374. https://doi.org/10.1098/rspb.2015.0374

Cecilia, H., Metras, R., Fall, A. G., Lo, M. M., Lancelot, R., & Ezanno, P. (2020). It's risky to wander in September: Modelling the epidemic potential of Rift Valley fever in a Sahelian setting. Epidemics, 33, 100409. https://doi.org/10.1016/j.epidem.2020.100409

Cheeseman, C. L., Little, T. W. A., Mallinson, P. J., Rees, W. A., & Wilesmith, J. W. (1985). The progression of bovine tuberculosis infection in a population of Meles meles in south-west England. Acta Zoologica Fennica[ACTA ZOOL. FENN.]. 1985.

Clifton-Hadley, R. S., Wilesmith, J. W., & Stuart, F. A. (1993). Mycobacterium bovis in the European badger (Meles meles): epidemiological findings in tuberculous badgers from a naturally infected population. Epidemiol Infect, 111(1), 9-19. https://doi.org/10.1017/s0950268800056624

Conlan, A. J. K., McKinley, T. J., Karolemeas, K., Pollock, E. B., Goodchild, A. V., Mitchell, A. P., Birch, C. P. D., Clifton-Hadley, R. S., & Wood, J. L. N. (2012). Estimating the hidden burden of bovine tuberculosis in Great Britain.

Fischer, E. A., van Roermund, H. J., Hemerik, L., van Asseldonk, M. A., & de Jong, M. C. (2005). Evaluation of surveillance strategies for bovine tuberculosis (Mycobacterium bovis) using an individual based epidemiological model. Prev Vet Med, 67(4), 283-301. https://doi.org/10.1016/j.prevetmed.2004.12.002

Gallagher, J., Monies, R., Gavier‐Widen, M., & Rule, B. (1998). Role of infected, non‐diseased badgers in the pathogenesis of tuberculosis in the badger. Veterinary Record, 142(26), 710-714.

Hartemink, N. A., Purse, B. V., Meiswinkel, R., Brown, H. E., de Koeijer, A., Elbers, A. R., Boender, G. J., Rogers, D. J., & Heesterbeek, J. A. (2009). Mapping the basic reproduction number (R(0)) for vector-borne diseases: a case study on bluetongue virus. Epidemics, 1(3), 153-161. https://doi.org/10.1016/j.epidem.2009.05.004

Kao, R. R., Gravenor, M. B., Charleston, B., Hope, J. C., Martin, M., & Howard, C. J. (2007). Mycobacterium bovis shedding patterns from experimentally infected calves and the effect of concurrent infection with bovine viral diarrhoea virus. J R Soc Interface, 4(14), 545-551. https://doi.org/10.1098/rsif.2006.0190

Little, T. W., Naylor, P. F., & Wilesmith, J. W. (1982). Laboratory study of Mycobacterium bovis infection in badgers and calves. The Veterinary Record, 111(24), 550-557.

M. H. Poola, V. E. O., A. R. Cromieb, B.W. Wickhamb, R. F. Veerkampa. (2005). To One Cow Survival and Fertility Evaluation for Irish Dairy and Beef Cattle.

Maher, P., Good, M., & More, S. (2008). Trends in cow numbers and culling rate in the Irish cattle population, 2003 to 2006. Irish veterinary journal, 61(7), 455. https://doi.org/10.1186/2046-0481-61-7-455

Rogers, L. M., Cheeseman, C. L., Mallinson, P. J., & Clifton‐Hadley, R. (1997). The demography of a high‐density badger (Meles meles) population in the west of England. Journal of Zoology, 242(4), 705-728. https://doi.org/10.1111/j.1469-7998.1997.tb05821.x
